# Effects of Spinal Manipulation and Dry Needling on Headache and Migraine: A Systematic Review of Randomized Controlled Trials

**DOI:** 10.3390/jcm15052084

**Published:** 2026-03-09

**Authors:** Rubén Maroto-García, Samuel Sánchez-Fernández, Germán Monclús-Díez, Sandra Sánchez-Jorge, Mónica López-Redondo, Marcin Kołacz, Dariusz Kosson, Juan Antonio Valera-Calero

**Affiliations:** 1 Department of Physiotherapy, Faculty of Nursery, Physiotherapy and Podiatry, Complutense University of Madrid, 28040 Madrid, Spain; rumaroto@ucm.es (R.M.-G.); samusa01@ucm.es (S.S.-F.); gmonclus@ucm.es (G.M.-D.); 2 Faculty of Health Sciences, Universidad Francisco de Vitoria, 28223 Madrid, Spain; monica.lopezredondo@ufv.es; 3 1st Department of Anaesthesiology and Intensive Care, Medical University of Warsaw, 02-005 Warsaw, Poland; marcin.kolacz@wum.edu.pl; 4 Division of Teaching, Department of Anaesthesiology and Intensive Care, Medical University of Warsaw, 02-005 Warsaw, Poland; dariusz.kosson@wum.edu.pl

**Keywords:** cervicogenic headache, dry needling, migraine, physiotherapy, spinal manipulation, tension-type headache

## Abstract

**Background/Objectives**: Cervical pain is defined as pain in the neck that may or may not radiate to one or both upper extremities and lasts at least one day. Headaches are within the spectrum of neck pain, defined as any painful sensation perceived in the head that can extend to the neck. They are classified as primary (migraines and tension headaches) or secondary (cervicogenic headaches) depending on their clinical presentation and associated symptoms. The objective of this review is to compare the effects of dry needling with and without spinal manipulative techniques versus the application of other physical therapy modalities. **Methods**: A systematic review was conducted searching articles compatible with the objectives of this study in PubMed, ScienceDirect, and Scopus databases using the search terms spinal manipulation, cervical manipulation, dry needling, headache, headaches, and migraine over the last five years and combined with the Boolean operators AND and OR. After screening, all studies underwent methodological quality assessments using the PEDro scale and qualitative synthesis for study design, patients’ characteristics, interventions, comparators, outcomes assessed and main results data. **Results**: Thirteen randomized clinical trials were selected. The quality of the studies is varied, with PEDro scale values ranging from six to eight. Dry needling and cervical manipulations have proven to be effective tools, compared to other interventions, in reducing pain and improving functionality in patients with headaches. **Conclusions**: Dry needling techniques and manipulations have shown significant effects on parameters related to pain, sensitivity, functionality, and general health in patients with headaches. However, future studies are necessary to more deeply analyze the long-term effects of both techniques.

## 1. Introduction

The International Headache Society defines headache as any painful sensation perceived in the head that may extend to the neck. This society classifies headaches as primary (i.e., migraine and tension-type headache (TTH)) or secondary (i.e., cervicogenic headache), based on their clinical presentation and associated symptoms [1].

Migraines are characterized by recurrent headache episodes with a throbbing or pulsatile sensation. The frequency, intensity, and duration of these episodes are variable, and the pain is generally localized unilaterally, exacerbated by physical activity, and accompanied by symptoms such as nausea and vomiting [2]. In some cases, transient and fully reversible neurological signs, known as migraine aura, may occur [1,3]. On the other hand, TTH usually presents with bilateral episodes, of a pressing nature and generally mild to moderate intensity, lasting from minutes to days [4]. The pain does not worsen with physical activity, nor is it associated with nausea, although photophobia or phonophobia may coexist. In contrast, cervicogenic headache is a type of secondary headache originating from a lesion in the cervical spine and its osseous, myofascial, and/or discal components [5]. It is characterized by unilateral pain that may present an intermittent or continuous course, is commonly associated with a decreased cervical range of motion and its symptoms are often provoked by specific neck movements, sustained postures, or digital pressure applied to the posterior regions of the neck [3,6,7].

Epidemiological evidence consistently shows that primary headaches are the most prevalent. TTH is the most prevalent headache disorder worldwide, affecting approximately 26–27% of the global population [8]. About two billion individuals suffered from TTH in 2021 [9], with a notable 37% increase in global prevalence from 1990 to 2019 [10,11]. Migraine, while less common than TTH, still represents a major global health burden, affecting approximately one billion individuals in 2021 [12], with women being affected twice as often as men (20.7% vs. 9.7%) [13]. Secondary headaches remain relatively uncommon, with prevalence estimates of 3.9% in the general population [14,15].

According to current clinical practice guidelines (CPGs) [16,17,18,19], the first-line management of migraine and other primary headaches remains predominantly pharmacological, relying on agents such as nonsteroidal anti-inflammatory drugs, non-opioid analgesics, beta-blockers, antidepressants, antiemetics, and serotonin receptor agonists [16,18,19]. In parallel, non-pharmacological interventions play a complementary and preventive role, encompassing both behavioral approaches (such as psychotherapy, sleep regulation, and relaxation training) and physical therapies (such as neuromodulation, biofeedback, transcutaneous electrical nerve stimulation, spinal manipulation (SM), acupuncture, and joint mobilization techniques). Among the latter, SM (defined as a high-velocity, low-amplitude thrust applied to a spinal segment with the aim of restoring joint mobility and reducing pain and disability [20,21]) and dry needling (DN, which involves the insertion of solid filiform needles into myofascial trigger points (MTrPs) to induce analgesic effects [22]) have gained increasing attention for their potential to modulate pain and improve musculoskeletal dysfunctions associated with a wide range of musculoskeletal conditions [23,24], including headache disorders [16,17,19]. However, the evidence supporting these interventions remains fragmented, with variations in protocols, frequency, and reported outcomes.

In fact, a CPG [17] suggests that interventions combining SM, mobilization, and exercise may benefit patients with migraine, TTH or cervicogenic headache. Recommended treatment frequencies typically range from one to two sessions per week for 8 weeks for migraine, with complementary exercises targeting the deep cervical flexors often included as part of multimodal care [25]. Nevertheless, while short-term improvements in pain and function have been reported, these recommendations are graded as weak to moderate due to heterogeneity in study designs, small sample sizes, and lack of standardized treatment parameters. Thus, despite its potential therapeutic value, the evidence supporting SM remains limited, with considerable variability in protocols, frequency, target regions, and outcome measures across studies [17].

Similarly, DN is considered a promising intervention within physiotherapy for the management of myofascial pain and headache disorders [26]. DN is primarily indicated for the inactivation of MTrPs in muscles commonly associated with referred pain patterns in the head and neck (such as the temporalis, upper trapezius, and sternocleidomastoid, among others [27]). Several studies have demonstrated reductions in headache frequency and intensity when DN is applied alone or in combination with manual therapy or exercise [26,28]. Nonetheless, as with SM, the level of evidence remains low to moderate, and no consensus has been reached regarding needle insertion depth, stimulation technique (superficial vs. deep), session frequency, or the inclusion of electrical stimulation modalities. Consequently, although the reported effects are promising, the existing literature is heterogeneous, and a critical review of the literature is warranted to establish standardized and reproducible DN protocols for headache management.

Although both SM and DN are frequently used to address cervical dysfunction and MTrPs within a musculoskeletal framework, the pathophysiology of headache disorders also involves broader neurobiological processes. Current models emphasize the role of trigeminovascular activation in headache generation, and trigemino-autonomic pathways may further contribute to symptom expression and modulation in some patients. In this regard, the growing interest in neuromodulatory targets such as the sphenopalatine/pterygopalatine ganglion (whose blockade has been considered a promising approach for the treatment of a variety of conditions such as post-dural puncture headache, migraine, trigeminal neuralgia, cluster headache and vasomotor rhinitis) reinforces the idea that musculoskeletal, nociceptive, and autonomic mechanisms may coexist rather than operate as mutually exclusive explanatory models [29]. Therefore, the reported neurophysiological effects of SM and DN [22,30] provide additional rationale for investigating these interventions in headache populations.

Previous reviews have evaluated DN and SM largely as separate therapeutic approaches. Reviews focused on DN have synthesized its effects in headache populations [26,28], whereas reviews of SM have mainly addressed specific headache entities such as cervicogenic headache or migraine [31,32]. However, to our knowledge, no recent review has integrated randomized controlled trials examining both interventions within the same synthesis, including studies in which DN and SM were applied in combination. In light of the aforementioned gaps in the literature, this review aimed to systematically review experimental studies applying DN, alone or in combination with SM, in the management of primary and secondary headaches in order to determine whether these interventions improve key clinical outcomes such as pain intensity, headache frequency, and related disability when compared with other commonly applied therapeutic approaches.

## 2. Materials and Methods

### 2.1. Study Design

This systematic review was conducted in accordance with the Preferred Reporting Items for Systematic Reviews and Meta-Analyses (PRISMA) guidelines [33] (Table S1). The search, study selection, methodological quality assessment and the qualitative synthesis were conducted by two members of the research group with the assistance of an experienced health science librarian. The study protocol is registered and publicly available in the Open Science Framework platform (https://doi.org/10.17605/OSF.IO/2JXZN, updated 30 October 2025).

### 2.2. Data Sources

Following the recommendation to consult at least three databases to ensure adequate coverage in systematic reviews [34], electronic searches were conducted in PubMed, Scopus, and ScienceDirect. Search strategies were based on the combination of MeSH terms and free terms combined with Booleans operators, following the PICO question (Population, Intervention, Comparison, and Outcomes):

**Population:** Adults suffering from tension-type headaches, cervicogenic headaches or migraines.

**Intervention:** DN alone or in combination with SM.

**Comparator:** Any other physiotherapeutic intervention or no intervention.

**Outcomes:** Headache intensity, frequency and duration, function or quality of life.

An example of the search strategy (used for PubMed) is as follows:

Filters: [Title/Abstract]#1 “Headache Disorders” [MeSH]#2 “Tension-Type Headache”#3 “Cervicogenic Headache”#4 Headaches#5 Headache#6 “Migraine Disorders” [MeSH]#7 Migraine#8 #1 OR #2 OR #3 OR #4 OR #5 OR #6 OR #7#9 “Dry Needling” [MeSH]#10 “Spinal Manipulation” [MeSH]#11 “Cervical Manipulation”#12 #9 OR #10 OR #11#13 #8 AND #12

### 2.3. Study Eligibility Criteria

The eligibility of the studies was determined according to predefined inclusion and exclusion criteria to ensure methodological rigor and relevance to the study objectives.

Inclusion criteria: Studies were eligible if they (1) examined the effects of DN or spinal/manipulative techniques on pain and functional outcomes, either as standalone interventions or in comparison with other physiotherapy approaches; (2) were published within the last five years (a decision supported by methodological guidance which emphasized that evidence syntheses may become outdated within a relatively short period [35], particularly in areas where new trials and updated evidence continue to emerge); (3) were written in English or Spanish; and (4) were designed as randomized controlled trials involving human participants.

Exclusion criteria: Studies were excluded if (1) they were not original peer-reviewed research (e.g., editorials, conference abstracts, or posters); (2) they involved participants under 18 years of age; (3) they compared the interventions with non-physiotherapeutic (medical) treatments; or (4) full text reports were not available.

### 2.4. Study Appraisal, Methodological Quality Assessment and Synthesis Methods

Mendeley Desktop v.1.19.8 for Mac OS (Glyph & Cog LLC, Petaluma, CA, USA) was used to manage and organize the search results obtained from the databases. Duplicate records were first removed. Subsequently, two independent reviewers screened the titles and abstracts to assess potential eligibility. Full-text articles were then evaluated to confirm inclusion. Any disagreements between reviewers were resolved through discussion; if consensus could not be reached, a third reviewer was consulted to make the final decision.

The methodological quality of the included trials was evaluated using the PEDro scale [36], a validated tool designed to appraise the internal validity and interpretability of randomized controlled trials. The scale comprises 11 items, of which the first (addressing external validity) is not included in the total score. The remaining ten items assess methodological criteria such as random allocation, concealed allocation, baseline comparability, blinding of participants, therapists, and assessors, completeness of follow-up, intention-to-treat analysis, between-group comparisons, and reporting of point estimates with measures of variability for at least one key outcome. According to established thresholds, total PEDro scores of 0–3 indicate poor quality, 4–5 fair quality, 6–8 good quality, and 9–10 excellent methodological quality [36].

Data extraction was performed using a standardized form based on the STARLITE guideline [37], including details on the study population, methodology (intervention, comparator, tasks, and muscles assessed), outcomes, and results.

## 3. Results

### 3.1. Study Selection Process

**Figure 1:**
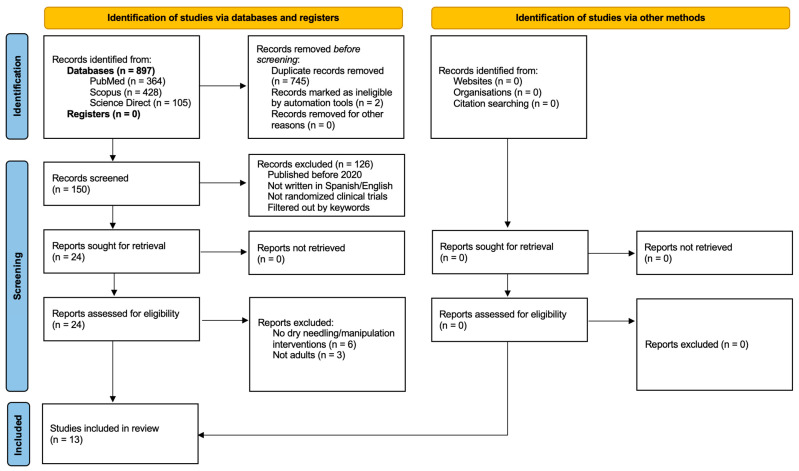
PRISMA 2020 flow-chart diagram.

The initial search identified 897 records. After applying filters and removing irrelevant results, 150 articles remained for title and abstract screening. Of these, 126 were excluded for not meeting the eligibility criteria or for including participants under 18 years of age. The remaining 24 studies underwent full-text assessment, during which nine duplicates were identified across databases. Ultimately, 13 randomized controlled trials met all inclusion criteria and were included in this review. The detailed selection process is illustrated in Figure 1.

### 3.2. Critical Appraisal and Risk of Bias

According to the PEDro scale appraisal, 12 of the included studies demonstrated high methodological quality and a low risk of bias [38,39,40,41,42,43,44,45,46,47,48,49], while one study showed moderate quality with a higher risk of bias [50]. Among these, nine trials were previously rated in the PEDro database [38,42,43,44,45,46,47,48,49,50], and the remaining four were independently assessed following the same criteria [39,40,41,47].

The mean PEDro score was 7.46, indicating good overall methodological quality. The most consistently fulfilled items were random allocation (item 1), concealed allocation (item 2), baseline comparability (item 3), adequate follow-up (item 7), between-group statistical comparisons (item 9), and reporting of point estimates with variability measures (item 10). Conversely, items 4 and 5, blinding of participants and therapists, were the least frequently achieved criteria. A detailed summary of total and item-level PEDro scores is presented in Table 1.

### 3.3. Synthesis of Clinical Characteristics

All included trials were conducted in adult patients diagnosed primarily with cervicogenic or migraine-type headaches, encompassing a total sample of 791 participants. Participants typically experienced recurrent, moderate-intensity headaches, either unilateral or bilateral, often accompanied by tenderness on palpation, and, across most studies, women represented the majority of participants [39,40,43,46,49,50].

### 3.4. Synthesis of Intervention Details

SMs were primarily applied to cervical and upper thoracic segments, whereas DN targeted cervicodorsal muscles. Both interventions were administered either independently or in combination with other physiotherapeutic approaches, and were commonly compared against placebo, manual therapy, mobilization techniques, or therapeutic exercise. The main outcomes assessed included headache frequency, intensity, duration, and disability level.

Across the selected trials, DN and high-velocity, low-amplitude (HVLA) manipulations were the primary techniques studied for the management of headaches and migraines. Six trials compared needling interventions with other approaches, including non-thrust manipulations [44], ischemic compressions [48], conventional physiotherapy [43], and placebo [40,43,48]. Among these, one study applied electro-DN [44], while another used superficial needling as a variation of the technique [40]. Three of the DN trials incorporated a control group [39,43,48].

A total of eight studies investigated manipulative techniques as the primary intervention. These were mainly cervical manipulations, compared with soft tissue mobilization [41], conventional physiotherapy [42], Mulligan techniques [38], usual care [49], myofascial release [45], non-thrust mobilizations/manipulations [44,46], or no treatment [50]. Some trials compared cervical versus thoracic manipulations [42], combined both regions [44], or applied thoracic manipulation alone [50]. Two of these studies also included a control group [38,45].

Finally, therapeutic exercise was incorporated in nearly all studies as a co-intervention, encompassing mobility, strengthening (isometric and concentric), stability, and stretching exercises [38,41,42,43,44,45,46,49,50].

### 3.5. Qualitative Synthesis

Table 2 summarizes participant characteristics, interventions, outcomes and results of the selected studies in this review.

#### 3.5.1. Pain and Pressure Sensitivity

Pain-related outcomes (intensity, frequency, duration, and pressure pain thresholds (PPTs)) were key measures across the included trials. Among the 13 studies, six used the visual analogue scale (VAS) to assess subjective pain or fatigue [18,38,39,41,42,48], while five employed the numeric pain rating scale (NPRS) [40,43,44,46,50]. Eight trials evaluated PPT using algometry [38,39,40,41,42,45,47,48].

All studies that assessed pain or sensitivity reported improvements from baseline, with nine trials demonstrating that DN and/or SM achieved greater reductions in headache frequency, duration, intensity, and pressure sensitivity compared to control or alternative interventions [39,40,42,43,44,45,47,49,50].

For instance, Nambi et al. [42] observed superior pain reduction following cervical manipulation compared with thoracic manipulation. In contrast, four studies found no significant between-group differences for pain or sensitivity outcomes [40,43,46,48]. Two additional trials reported either non-supportive results for the isolated interventions [41], or greater improvements with alternative techniques such as Mulligan mobilizations [38].

#### 3.5.2. Functional Capacity and General Health

Functional outcomes and measures of disability or general health were assessed through a variety of validated instruments. The Headache Impact Test (HIT) [38,41,42,45,46,49] and the Neck Disability Index (NDI) [38,41,42,43,44,45,46,50] were the most frequently employed tools to quantify the impact of headaches and neck pain on daily activities. McDevitt et al. [50] additionally used the Headache Disability Inventory (HDI), while Rist et al. [49] used the Migraine Disability Assessment Scale (MIDAS) to evaluate migraine-related disability.

Cervical mobility was examined in several studies using goniometric measures of active and passive cervical range of motion (CROM) [38,40,43,47], and upper cervical dysfunction was assessed via the cervical flexion–rotation test (CFRT) [38,40,42]. The craniocervical flexion test (CCFT) was implemented once to assess deep cervical flexor performance [43].

Patient-reported outcomes related to quality of life and perceived improvement were also frequently included. Two studies used the EQ-5D to evaluate overall quality of life [38,42], whereas a single study [49] applied the Migraine-Specific Quality of Life Questionnaire (MSQL). The global rating of change (GROC) was used in four studies [39,44,46,50], while a single study used the patient acceptable symptom state (PASS) to determine symptom acceptability thresholds to capture patients’ perception of clinical change [46].

Across the reviewed trials, most interventions produced improvements in disability, functionality, range of motion, and quality of life compared to baseline. Eight studies reported results favoring DN and/or SM for these outcomes [39,40,42,43,44,45,48,50].

Nambi et al. [42] observed superior gains in disability scores, CFRT results, and quality-of-life measures with cervical manipulations compared to thoracic manipulations. Conversely, three studies found non-significant changes across tools such as NDI, HDI, HIT, GROC, FRT, and PASS [40,46,50]. Finally, two of the studies reported results that either did not support isolated interventions [41] or showed greater benefits with the application of other techniques [38].

#### 3.5.3. Other Variables

In addition to pain and functional outcomes, several studies assessed secondary variables. Three trials evaluated changes in medication intake [44,47,49], one examined sternocleidomastoid muscle thickness changes using ultrasound [47], one assessed participant recruitment, retention, adherence, and the incidence of adverse effects [49] and one measured the elastic modulus and the area of the MTrPs in the sternocleidomastoid muscle [48].

Regarding these secondary outcomes, Dunning et al. [44] reported a significant reduction in medication intake favoring DN combined with SM, while Rezaeian et al. [47] observed greater increases in sternocleidomastoid (SCM) thickness in the DN group. Conversely, Togha et al. [48] found no significant short-term differences between groups in MTrP area or length. Notably, DN and manipulation were associated with more adverse effects than comparison techniques in some studies [48,49].

## 4. Discussion

The present review aimed to examine the effects of DN and SM (applied either individually or in combination) on patients with primary and secondary headaches, and to compare their outcomes with those of other commonly used physiotherapeutic interventions. The literature synthesis summarizes the results of thirteen randomized controlled trials with good methodological quality, encompassing a total of 791 participants with migraine, TTH, or cervicogenic headache. Overall, the evidence suggested that both DN and SM are effective in reducing pain intensity, frequency, and pressure sensitivity (nine articles reported favorable results in terms of pain parameters and pressure sensitivity [39,40,42,43,44,45,47,49,50]), as well as improving functional capacity, disability scores, and quality of life (eight articles showed favorable results regarding functionality, disability, and patients’ quality of life [39,40,42,43,44,45,47,50] and two studies obtained favorable results regarding medication intake and muscle thickness changes [44,47]).

Across the included studies, most interventions were applied within multimodal programs incorporating mobility, strengthening, stability, and stretching exercises, which may have influenced the observed outcomes [38,41,42,43,44,45,46,49,50]. Despite this variability, the consistency of improvements across pain and functional parameters reinforces the clinical relevance of DN and SM as effective short-to-mid-term (up to 6-month follow-up) interventions based on studies with moderate-to-good methodological quality.

Therefore, from a clinical perspective, the findings indicate that multimodal treatment approaches integrating DN directed to active MTrPs in craniocervical muscles frequently associated with headache presentations (e.g., upper trapezius, sternocleidomastoid and suboccipital muscles), SM applied to the cervical and/or thoracic spine, and mobility, strengthening, stabilization and stretching therapeutic exercises may offer greater clinical benefits than the isolated use of either technique. Such a combined approach may facilitate short-to-mid-term improvements in pain, headache frequency, disability, and cervical dysfunction.

### 4.1. Manipulations Compared to Other Physiotherapy Techniques

The effectiveness of SM was evaluated against several physiotherapeutic comparators in patients with cervicogenic headache [38,41,42,44,46,50], TTH [45], and migraine [49]. The control or comparison interventions included Mulligan mobilization [38], instrument-assisted soft tissue mobilization added to SM and exercise [41], thoracic SM and conventional physiotherapy treatment [42], non-thrust mobilization combined with exercise [44], myofascial release plus exercise and exercise alone [45], mobilization combined with a home exercise program [46], and enhanced usual care in a multimodal chiropractic trial in migraine [49]. Most manipulation protocols targeted the upper cervical spine, particularly in cervicogenic headache and TTH [38,41,42,44,45,46], whereas thoracic manipulation was specifically evaluated in cervicogenic headache either as a comparator to cervical SM [42] or combined with thoracic mobility exercise versus no treatment [50].

Across headache types, the most consistent comparative benefit of manipulation was observed in cervicogenic headache. In this population, cervical SM generally produced greater improvements in headache frequency, pain intensity, disability, and neck-related outcomes than thoracic SM or conventional physiotherapy [42], and it also outperformed no treatment when thoracic SM was assessed in a crossover design [50]. However, superiority was not universal across all manual comparators: Mulligan mobilization achieved better short- and long-term outcomes than cervical SM in chronic cervicogenic headache [38], while another pragmatic trial found that manipulation and mobilization combined with home exercise produced comparable improvements, with no significant between-group differences [46]. This suggests that, in cervicogenic headache, SM is often effective, but its comparative advantage depends on the specific manual technique against which it is tested.

In TTH, evidence was limited to one trial [45], but findings favored upper cervical SM combined with exercise over both myofascial release plus exercise and exercise alone. The SM group was the only one to show significant and maintained improvements across all main variables, including headache frequency, headache and neck pain intensity, disability, and pressure pain thresholds at 3-month follow-up [45]. Compared with cervicogenic headache, where results were more heterogeneous depending on the comparator [38,46], the TTH evidence appears more consistently favorable to SM, although it is based on a single study and therefore should be interpreted cautiously.

In migraine, the evidence was even more limited and less attributable to SM specifically. The only included trial [49] assessed multimodal chiropractic care plus enhanced usual care versus enhanced usual care alone. Although this multimodal intervention produced greater reductions in migraine days and disability, the treatment package included not only manipulation and mobilization but also soft tissue techniques, exercise, posture correction, relaxation, ergonomic advice, and education [49]. Therefore, unlike cervicogenic headache and TTH studies where the role of SM was more directly tested, the migraine evidence supports a multimodal chiropractic approach rather than isolated SM.

Intervention combinations also appear relevant when interpreting the magnitude and persistence of treatment effects. Studies in which manipulation was combined with additional active or adjunctive components tended to show larger and more sustained improvements than those comparing thrust and non-thrust manual approaches delivered alongside similar exercise programs. For example, adding instrument-assisted soft tissue mobilization to cervical SM and exercise resulted in superior outcomes to SM and exercise alone in cervicogenic headache, especially at 6-month follow-up [41]. Similarly, cervical and upper thoracic thrust manipulation combined with electrical DN produced greater reductions in headache intensity, frequency, disability, and medication use than non-thrust mobilization plus exercise at 3 months [44]. In contrast, when both groups received similar exercise-based co-interventions, differences between SM and mobilization were attenuated or disappeared [46].

Regarding the time course of effects, manipulation-related benefits were most consistently observed in the short term, but some studies also showed maintenance at medium- and long-term follow-up, particularly when manipulation formed part of a multimodal program. Short-term improvements were reported in TTH [45], cervicogenic headache [44,50], and migraine within multimodal chiropractic care [49]. More durable benefits, up to 3 or 6 months, were especially evident in CGH trials involving cervical SM combined with exercise and adjunctive soft tissue approaches [41,42], as well as in the TTH trial combining manipulation with exercise [45]. By contrast, when manipulation was compared against another active manual treatment delivered with similar co-interventions, long-term superiority was less clear [38,46].

Overall, the available evidence suggests that SM may be most beneficial in cervicogenic headache, where it frequently outperforms thoracic manipulation, conventional physiotherapy, or no treatment, although not necessarily other skilled manual approaches such as Mulligan mobilization [38,42,50]. In TTH, the evidence also favors manipulation, especially when combined with exercise [45], whereas in migraine the available data support a broader multimodal chiropractic model rather than the isolated effect of manipulation [49]. Thus, comparative interpretation across headache types indicates a stronger and more direct evidence base for manipulation in cervicogenic headache, a promising but limited basis in TTH, and only indirect support in migraine. In addition, cervical SM appears more effective than thoracic manipulation or no treatment for reducing pain intensity, frequency, and related disability. These outcomes may be explained by its ability to enhance afferent input from cervical joint receptors, thereby modulating alpha motor neuron excitability and improving muscular coordination [51]. The high mobility of cervical vertebrae allows for greater activation of deep neck muscle receptors, a mechanism not elicited by thoracic manipulation [52]. Meta-analytic evidence supports these findings, indicating that cervical manipulation can reduce migraine pain and headache frequency [31], potentially through autonomic regulation and descending inhibitory pathways [53]. Nevertheless, these benefits are generally short-term and of small effect size, as shown in broader reviews of TTH and cervicogenic headache [54].

### 4.2. Dry Needling Compared to Other Physiotherapy Techniques

Several studies evaluated the effects of DN compared with sham procedures, passive control, or other physiotherapeutic interventions for the treatment of cervicogenic headache, migraine, and TTH [39,40,43,44,47,48]. The DN techniques investigated included deep trigger-point DN [39,43,47,48], superficial DN [40], and perineural electrical DN combined with SM [44]. Comparison groups included sham DN [40,43,47], passive control or no intervention [39,48], ischemic compression [48], conventional physiotherapy treatment (CPT) [43], and non-thrust mobilization combined with exercise [44].

Overall, most studies reported reductions in headache-related pain, frequency, duration, disability, or pressure pain sensitivity after DN, particularly when compared with sham or passive control conditions [39,40,43,47,48]. In TTH, Monti-Ballano et al. [39] found that DN reduced pain intensity, the number of active MTrPs, medication use, and improved perceived clinical change compared with passive control. In cervicogenic headache, superficial DN applied to trigeminal nerve terminal branches produced immediate improvements in pain and cervical mobility compared with sham DN, although pressure pain thresholds did not differ significantly between groups [40]. In migraine, Rezaeian et al. [47] reported greater reductions in headache frequency, intensity, duration, and drug consumption after DN than after sham DN, together with improvements in pressure pain thresholds and cervical range of motion.

Comparisons against other active physiotherapy interventions yielded a more nuanced pattern. In cervicogenic headache, Dunning et al. [44] reported that perineural electrical DN combined with SM produced greater short- and medium-term improvements in headache intensity, frequency, duration, disability, medication cessation, and perceived recovery than non-thrust mobilization plus exercise. Likewise, Mousavi-Khatir et al. [43] found that adding DN to conventional physiotherapy led to greater improvements in pain, disability, cervical range of motion, and deep neck flexor performance than sham DN plus physiotherapy or physiotherapy alone. By contrast, Togha et al. [48] observed that both DN and ischemic compression improved headache intensity, frequency, duration, and pressure pain thresholds compared with passive control, but no significant differences were found between the two active interventions. Therefore, although DN often performed better than sham or no treatment, its superiority over other active manual techniques was less consistent.

Comparative interpretation across headache types also suggests some differences. In cervicogenic headache [40,43,44,48], DN was studied using a wider range of approaches, from superficial trigeminal-field needling to deep MTrP protocols and electrically stimulated perineural techniques, generally with favorable results but substantial clinical heterogeneity. In contrast, the evidence in TTH [39] and migraine [47] was more limited in number of studies, although both showed beneficial effects of DN versus inactive or sham comparators. This means that the apparent consistency of results is stronger when DN is compared with non-active conditions than when it is tested against other active physiotherapy interventions.

Regarding functional and secondary outcomes, DN was also associated with improvements in cervical range of motion, disability, perceived recovery, and some neuromuscular variables [40,43,44,47]. Monti-Ballano et al. [39] reported better perceived clinical improvement with DN, whereas Mousavi-Khatir et al. [43] found greater gains in deep cervical flexors performance when DN was added to conventional physiotherapy. Rezaeian et al. [47] additionally reported increased sternocleidomastoid muscle thickness after DN in migraine, and Dunning et al. [44] found reduced medication intake in the electrical DN group. In Togha et al. [48], both DN and ischemic compression reduced trigger-point area compared with passive control, but neither intervention showed clear superiority over the other in biomechanical ultrasound measures.

When the time course of effects is considered, DN-related benefits were observed mainly in the short term, although some studies also showed maintenance at 1 to 6 months, particularly when DN was incorporated into multimodal protocols [43,44,47]. Immediate effects were reported after a single superficial DN session in cervicogenic headache [40], whereas short programs of 3–4 sessions were used in TTH, migraine, and cervicogenic headache trigger-point studies [39,47,48]. More intensive multimodal approaches extended over 4–5 weeks and up to eigght sessions [43,44]. This variability in dosage, treatment duration, needling approach, and co-interventions limits direct comparison across studies and may partly explain differences in the magnitude and persistence of effects.

Overall, DN interventions appear to be consistently more effective than sham or passive control in reducing headache-related pain and disability across cervicogenic headache, migraine, and TTH [39,40,43,47,48]. Their comparative advantage over other active physiotherapy techniques is less uniform, but may be greater when DN is delivered within multimodal protocols or combined with electrical stimulation [43,44]. Although the current evidence suggests that DN is a useful option for short-term symptom relief across headache types (while its superiority over other active manual therapies remains context-dependent [23,55]), the evidence is limited by the small number of studies within each headache subtype, heterogeneity in DN protocols and comparators, and variability in follow-up duration and outcome measures.

### 4.3. Manipulations vs. Dry Needling

Evidence from recent meta-analyses suggests that DN is supported by higher-quality evidence than SM, particularly regarding short-term pain reduction, whereas the evidence for SM remains limited and inconsistent, especially over medium- and long-term follow-up periods [23,32,53,55]. Direct comparisons between SM and DN were not available among the randomized trials included in this review. Therefore, any comparison between both approaches must be interpreted indirectly and with caution, taking into account the heterogeneity in headache subtype, treatment dosage, comparator groups, co-interventions, and follow-up duration across studies [38,39,40,41,42,43,44,45,46,47,48,49,50].

Both SM and DN were associated with improvements in headache-related outcomes, but the pattern of evidence differed between interventions. Studies on DN more consistently showed favorable results when compared with sham or passive control conditions, particularly for pain intensity, headache frequency, disability, and pressure pain sensitivity [39,40,43,47,48]. By contrast, SM also showed beneficial effects, especially in cervicogenic headache, but its comparative superiority appeared more dependent on the intervention against which it was tested. In some studies, cervical SM outperformed thoracic manipulation, conventional physiotherapy, or no treatment [42,50], whereas in others it showed similar or smaller effects than other active manual approaches such as Mulligan mobilization or mobilization combined with exercise [38,46].

Comparative interpretation across headache types also suggests some differences. The evidence for SM was more concentrated in cervicogenic headache and was generally stronger in this subgroup than in migraine or TTH, where fewer studies were available and manipulation was often embedded within broader treatment packages [45,49]. In contrast, DN showed favorable findings across cervicogenic headache, migraine, and TTH, although the number of studies within each subtype remained limited and the protocols were highly variable [39,40,43,47,48]. Thus, DN appeared to show a more uniform direction of benefit across headache populations, whereas the effects of SM seemed more condition- and comparator-dependent.

The role of treatment combinations is particularly relevant when interpreting the relative contribution of each technique. For SM, several findings suggest that outcomes may be more favorable when manipulation is integrated into multimodal programs rather than applied in isolation, as shown by the better results obtained when it was combined with instrument-assisted soft tissue mobilization [41] or when compared within exercise-based protocols [42,45]. Similarly, DN also appeared to yield larger or more sustained effects when delivered as part of multimodal care, such as when added to conventional physiotherapy [43] or combined with SM and electrical stimulation [44]. In this regard, the trial by Dunning et al. [44] is especially relevant, since the combination of upper cervical/thoracic thrust manipulation and perineural electrical DN was superior to non-thrust mobilization plus exercise for headache intensity, frequency, disability, medication use, and perceived recovery at 3 months. Although the study design does not allow the specific contribution of SM and DN to be separated, it suggests that combining both techniques within a multimodal program may enhance short- to medium-term outcomes.

Differences in treatment dosage and follow-up should also be considered. DN protocols ranged from single-session immediate-effect interventions [40] to short programs of 3–4 sessions [39,47,48] and multimodal regimens of up to eight sessions over 4–5 weeks [43,44]. SM protocols also varied substantially, from brief thoracic manipulation programs [50] to cervical protocols delivered 3–4 times per week over 4 weeks, often combined with exercise, heat, or additional manual therapy [38,41,42]. Across both intervention types, beneficial effects were more consistently reported in the short term, whereas evidence for medium- and long-term superiority was less uniform and often depended on the use of co-interventions [41,42,43,44,45,47].

Therefore, the available evidence does not allow a confident conclusion that either DN or SM is clearly superior to the other. Rather, both interventions appear to provide potential clinical benefit, particularly in the short term, but their effects seem to be influenced by headache subtype, comparator intervention, treatment dosage, and whether they are delivered alone or as part of a multimodal physiotherapy program. At present, the most robust interpretation is that DN may show more consistent benefits over sham or passive controls, whereas SM may be especially useful in cervicogenic headache but with more variable results when compared with other active manual approaches. Direct head-to-head trials are needed to determine their relative efficacy more clearly.

### 4.4. Safety Considerations and Report of Adverse Events

Adverse-event reporting across the included trials was heterogeneous and often limited in detail, although most studies explicitly stated whether complications occurred. Overall, no serious adverse events were reported in the included randomized trials. For DN, the most commonly described adverse effects were mild and self-limited post-needling soreness [39,43,44] and, less frequently, mild bruising [44]. In contrast, trials evaluating SM generally reported no serious adverse events [41,42,46,50], although adverse-event surveillance was often described only briefly. The migraine trial evaluating multimodal chiropractic care reported a higher number of non-serious adverse events in the active treatment group than in enhanced usual care, but no serious treatment-related complications were observed [49]. Therefore, the available trial evidence suggests that both interventions were generally well tolerated within the studied protocols, but the inconsistency and limited granularity of adverse-event reporting preclude firm conclusions regarding comparative safety.

Regarding cervical SM specifically, a balanced safety interpretation is warranted. Serious neurovascular complications such as cervical artery dissection appear to be rare, but the relationship between cervical high-velocity techniques and dissection remains debated in the literature [56]. Current evidence suggests that the observed association is difficult to interpret because of bias and confounding, particularly given that neck pain and headache may themselves represent early manifestations of dissection. At the same time, contemporary cervical assessment frameworks emphasize that vascular pathologies of the neck, although rare, should be considered during clinical reasoning before cervical manual therapy. Accordingly, cervical manipulation in headache populations (especially when vascular comorbidity or atypical clinical features are suspected) should be preceded by careful screening, clinical judgment, and appropriate risk stratification [57].

### 4.5. Limitations

A limitation of this review is that the search was restricted to three databases and to studies published within the last five years. Although the search strategy was designed in accordance with published methodological guidance suggesting and considering that including additional databases does not necessarily improve the quality of the evidence synthesis (as it may mainly increase duplicate retrieval and the identification of studies from sources with more variable editorial standards), this methodological decision may have increased the risk of missing potentially relevant studies indexed elsewhere. Similarly, the restriction to results published within the last 5 years may have increased the risk of missing potentially relevant studies.

Due to the heterogeneity of the studies, it was not possible to perform a quantitative analysis. This literature review presents limitations due to the small number of studies that met the inclusion criteria. Only articles in Spanish and English were included, so there may be studies in other languages providing relevant information that could not be included due to language limitations. Another limitation affecting the generatability of results is the small sample size in some of the included studies [39,40,48].

In most of the revised studies, additional exercise programs were applied to all treatment groups, which may have generated some bias, altering the effects obtained by the techniques under evaluation [38,41,42,43,44,45,46,49,50]. Moreover, it was not possible to perform long-term follow-ups to assess whether the effects and results obtained through the interventions persisted over time. The perception of pain, like other relevant variables in this review, is inherently subjective and can be exaggerated or minimized by the patients. Due to this, another limitation was the lack of objective measurements not dependent on participants’ perceptions.

## 5. Conclusions

Considering the results of this qualitative synthesis, DN and SM may represent potentially useful short-term interventions for reducing pain intensity, headache frequency, and disability in patients with primary and secondary headaches, particularly when integrated within multimodal physiotherapy programs. However, the current evidence base is limited by considerable heterogeneity in intervention protocols, comparators, follow-up duration, and outcome reporting, as well as by small sample sizes and the absence of significant between-group differences in several studies. Therefore, the available data do not allow firm conclusions regarding the consistent superiority, optimal application, or long-term efficacy of either technique. Future high-quality randomized controlled trials with standardized protocols, clearer intervention reporting, objective outcome measures, and extended follow-up periods are needed to better define the clinical role and optimal treatment parameters of DN and SM in headache management.

## Figures and Tables

**Table 1:** Methodological quality assessment based on the PEDro scale.

Article	PEDro Scale Score (Items & Total)											
	1	2	3	4	5	6	7	8	9	10	11	Total
Monti-Ballano et al. [39]	1	1	1	1	0	0	1	1	1	1	1	8/10
Porter et al. [40]	1	1	1	1	0	0	1	1	1	1	1	8/10
Nambi et al. [41]	1	1	1	1	0	0	1	1	1	1	1	8/10
Nambi et al. [42]	1	1	1	1	0	0	1	1	1	1	1	8/10
Nambi et al. [38]	1	1	1	1	0	0	1	1	0	1	1	7/10
McDevitt et al. [50]	1	1	1	1	0	0	0	1	0	1	1	6/10
Mousavi-Khatir et al. [43]	1	1	1	1	0	0	1	1	1	1	1	8/10
Dunning et al. [44]	1	1	1	1	0	0	1	1	1	1	1	8/10
Rist et al. [49]	1	1	1	1	0	0	0	1	1	1	1	7/10
Corum et al. [45]	1	1	1	1	0	0	1	1	0	1	1	7/10
Lerner-Lentz et al. [46]	1	1	1	1	0	0	0	1	1	1	1	7/10
Rezaeian et al. [47]	1	1	1	1	1	0	1	1	0	1	1	8/10
Togha et al. [48]	1	1	1	1	0	0	1	1	0	1	1	7/10

Legend of PEDro scale items: (1) eligibility criteria specified, (2) random allocation, (3) concealed allocation, (4) baseline comparability, (5) blinding of subjects, (6) blinding of therapists, (7) blinding of raters, (8) ≥85% follow-up for at least one key outcome, (9) intention-to-treat analysis, (10) between-group statistical comparisons, and (11) provision of point estimates with measures of variability.

**Table 2:** Synthesis of results: Study design, participants, intervention, comparators, outcomes assessed and results.

Reference	Participants	Interventions	Comparators	Outcomes	**Results**
Monti-Ballano et al. [39] Randomized controlled trial, single-blind, two parallel groups	**n = 32** Profile: Adults with TTH (episodic/chronic) diagnosed by neurologist according to ICHD-3; required ≥1 active MTrP in muscles referring pain to the head/face. Mean age 39.1 ± 12.7 years; 75% women; mean headache frequency 13.4 ± 10.2 days/month.	**DN (n = 16)** DN of active MTrPs in craniocervical muscles associated with TTH (upper trapezius, splenius capitis/cervicis, semispinalis, rectus capitis posterior major, obliquus capitis superior/inferior, occipitofrontalis (anterior/posterior), temporalis, masseter, sternocleidomastoid, zygomaticus major, levator scapulae). Three treatment sessions; total number of active MTrPs per subject distributed across sessions (each MTrP treated only once); in-and-out pistoning in large muscle bellies without nearby dangerous structures and bidirectional rotation technique in flat muscles and those near neurovascular structures; aim to elicit local twitch responses at each MTrP.	**Passive Control (n = 16)** No DN or other physiotherapy intervention during study period. Participants continued their usual headache medication.	Pain intensity: VAS. Total number of active MTrPs: palpation + algometry. Perceived clinical change: GROC. Change in headache medication use. All outcomes assessed pre- and post-treatment.	DN group showed a significant reduction in total number of active MTrPs compared with its own baseline (mean pre–post difference −4.5 points; 95% CI 0.25 to 8.75; *p* = 0.039); no significant change in control group. For pain intensity, within-group analysis showed a significant VAS reduction only in the DN group (*p* = 0.008); general linear model with medication change as covariate showed a significant post-treatment between-group difference favoring DN (mean VAS difference control–DN = 23.45 mm; 95% CI 1.89 to 45.01; *p* = 0.034). GROC was markedly better in the DN group compared with control (mean 3.94 ± 2.08 vs. −0.38 ± 2.28; *p* < 0.001), indicating clinically relevant improvement. Headache medication use post-treatment was lower in the DN group than in controls (*p* = 0.009). No serious adverse events were reported; only mild, self-limited post-needling soreness (upper trapezius, masseter, sternocleidomastoid) resolving within 48 h without treatment.
Porter et al. [40] Randomized controlled trial, parallel-group, participant- and assessor-blinded	**n = 30** Profile: Adults (≥18 years) with CGH; ≥1 headache per week for ≥3 months; unilateral headache pain; pain aggravated by neck movements or sustained postures; provocation by joint play C0-C4 and/or positive flexion–rotation test (FRT); headache or neck pain intensity ≥2/10.	**Superficial DN (n = 17)** Superficial DN targeting the innervation fields of the trigeminal nerve’s terminal branches bilaterally (supraorbital, infraorbital, mental, auriculotemporal nerves), regardless of local symptoms or tenderness. Participants in supine position; 0.18 × 15 mm needles inserted cutaneously/subcutaneously to a depth sufficient to secure the needle in the skin. Four rounds of 3–4 needle rotations per needle were applied to enhance the sensory effect. Total treatment time 5–7 min including compression/hemostasis. Single treatment session; immediate effects only were evaluated.	**Sham DN (n = 13)** Same set-up and standardized script as DN group (supine position, eyes closed, guide tube contact), but the needle was bent up inside the guide tube so that it did not pierce the skin. Guide tube pressure and treatment time were matched to the DN group to mimic the procedure without skin penetration. No other physiotherapy intervention was provided during the study period.	Pain intensity: NPRS. Cervical AROM of the most provocative movement: measured with a CROM device. CFRT: right and left, degrees, using a motion detection device. PPT: right and left supraorbital nerve; right and left greater occipital nerve, measured with a handheld algometer. All outcomes assessed at baseline and immediately post-treatment.	Compared with sham DN, superficial DN produced statistically and clinically meaningful immediate improvements in pain and cervical mobility. NPRS decreased more in the DN group (post 1.7 ± 1.1 vs. 3.7 ± 1.7; between-group mean difference 2.1 points; 95% CI 1.0 to 3.1; *p* < 0.001; d = 1.40), exceeding the MCID. AROM of the most provocative cervical movement increased more after DN (post 60.1 ± 15.3° vs. 42.2 ± 14.7°; mean difference 18.0°; 95% CI 6.9 to 25.3; *p* = 0.006; d = 1.17). Between-group differences for right and left FRT were not statistically significant (*p* = 0.137), but mean changes of about 7.0–8.8° (d = 0.57–0.70) met published MDC/MCID thresholds, suggesting potential clinical relevance. PPT values over the supraorbital and greater occipital nerves did not differ significantly between groups (*p* = 0.052–0.187), although effect sizes were moderate to large (d ≈ 0.53–0.88). Within the DN group, immediate pre–post improvements were significant for NPRS, FRT (right and left), and AROM; PPT did not change significantly. Sham group showed smaller, non-significant changes. Authors conclude that superficial DN of the trigeminal innervation field produces immediate, clinically meaningful improvements in pain and cervical mobility in CGH, although results are limited by small sample size and short-term follow-up.
Nambi et al. [41] Randomized, single-blind, active-controlled trial with two parallel groups	**n = 64** Profile: Adults (>18 years) with unilateral CGH >3 months, diagnosed according to ICHD-3 (11.2.1) criteria; headache intensity ≥3/10; headache attributed to cervical spine dysfunction; reduced cervical motion on flexion–rotation test (FRT) with neck pain followed by headache; neck stiffness and movement restriction; no cervical fracture, osteoporosis, deformity or abnormalities on cervical imaging. Mean age ≈ mid-30s; 47–41% males; majority had associated neck pain (81–88%).	**SMT + ISM (n = 32)** Ten min hot hydrocollator pack to cervical region (base of neck and upper shoulders) and supervised neck isometric exercises three times per day (multi-directional resisted holds of 10 s × 15 repetitions), four sessions/week for 4 weeks, plus instructions to continue exercises after 4 weeks. + Cervical HVLAT per Peterson & Bergman protocol, primarily targeting upper cervical (C1–C2) segments; patient supine, therapist at head, bimanual pre-manipulative rotation 30–45°, thrust directed first away from and then toward the side of pain, within WHO safety guidelines. + Instrument-assisted soft tissue mobilization using an M2T blade: patient seated leaning forward, massage cream applied; restricted soft-tissue areas in cervical region identified; fascial release performed at ~45° angle along muscle fiber direction over levator scapulae, sternocleidomastoid, scalenes, upper trapezius (origin to insertion bilaterally, ~8 min), as well as longissimus capitis, splenius capitis, semispinalis capitis and suboccipital muscles in a centripetal direction, repeated three times; procedure stopped if adverse sensations occurred. Treatments delivered by experienced manual therapists, four sessions/week for 4 weeks.	**SMT (n = 32)** Same protocol of cervical HVLAT (assessment of cervical joint dysfunction each visit; manipulation directed primarily at C1-C2 with rotation away from and then toward pain in supine), same 10 min hot pack and identical neck isometric exercise program and instructions as in SMT + ISM group. No instrument-assisted soft tissue mobilization was added. Frequency and duration of treatment matched (four sessions/week for 4 weeks). Participants were asked not to receive other treatments during the study period.	CGH and neck pain frequency: number of CGH and neck pain days over 4 weeks. Pain intensity: VAS. Disability: HIT-6 and NDI. Neck pain threshold: PPT over upper trapezius trigger point using digital algometer. Health-related quality of life: EuroQol-5D. All outcomes measured at baseline, after 4 weeks, and at 6-month follow-up.	Both groups showed statistically significant improvements over time in the primary and secondary outcomes, but SMT + ISM generally produced larger and more sustained changes. For CGH frequency, SMT decreased from 15.6 ± 1.5 to 10.5 ± 1.1 days/4 weeks at 4 weeks and to 3.5 ± 0.5 at 6 months, while SMT + ISM decreased from 16.1 ± 1.4 to 6.2 ± 0.9 and to 1.8 ± 0.4, with between-group differences favoring SMT + ISM at 4 weeks and 6 months (*p* = 0.001). CGH pain intensity (VAS) decreased in SMT from 6.8 ± 0.9 to 4.5 ± 0.6 and 0.8 ± 0.2, and in SMT + ISM from 7.1 ± 1.1 to 3.1 ± 0.4 and 0.3 ± 0.3, again with superior reductions in SMT + ISM (*p* = 0.001 at 4 weeks and 6 months). CGH disability (HIT-6) improved in both groups but was lower (better) in SMT + ISM at both follow-ups (*p* = 0.001). Secondary neck-related outcomes (neck pain frequency and intensity, NDI, FRT, and quality of life) all improved significantly in both groups; mixed-model ANOVA showed significant group × time interactions favoring SMT + ISM for most variables, and standardized mean differences indicated greater percentage improvement in the SMT + ISM group. Neck pain threshold did not differ between groups at 4 weeks but was significantly higher (better) in SMT + ISM at 6 months (*p* = 0.016). No adverse events or serious complications were reported during or after the interventions. The authors conclude that adding instrument-assisted soft tissue mobilization to SM and exercise yields superior long-term outcomes compared with SM and exercise alone in unilateral CGH.
Nambi et al. [42] Randomized controlled trial, three parallel groups	**n = 96** Profile: Adults 18–60 years with unilateral or bilateral CGH >3 months, diagnosed by physician according to ICHD-3 (11.2.1) criteria; pain intensity ≥3/10 on VAS; CGH arising from neck pain followed by headache; limited cervical range of motion, neck stiffness and cervical spine musculoskeletal disorders. Mean CGH duration ≈ 6–7 years; majority with associated neck pain (84–88%) and unilateral headache (78–84%).	**Cervical SMT (n = 32)** HVLAT cervical spine manipulation at C1–C2 in supine “cradle hold” position; head positioned in extension, PA shift, ipsilateral side-bend and contralateral side-shift; single HVLAT rotation thrust first toward the non-painful, then the painful side; up to two attempts per session if no cavitation; three sessions/week for 4 weeks (12 sessions). + Ten min hot hydrocollator pack to neck/upper back before treatment and a home/clinic program of neck isometric exercises (multi-directional resisted holds 10 s × 15 reps, 3/day) plus static stretching of upper trapezius, levator scapulae, scalenes and sternocleidomastoid (30 s × 3 reps); asked to continue exercises after 4 weeks.	**Thoracic SMT (n = 32)** HVLAT thoracic spine manipulation at T1–T2 in supine, arms folded across chest, therapist contact over transverse processes and sternum with AP thrust; up to two attempts per session; three sessions/week for 4 weeks. + Same 10 min hot pack and identical neck isometric and stretching exercise program. **CPT (n = 32)** Massage therapy to cervical region for 15 min (circular kneading with fingertips along C7–C1, then suboccipital and cervical paravertebral muscles bilaterally in prone), plus the same 10 min hot pack and identical neck isometric and stretching exercise program.	CGH and neck pain frequency: number of CGH and neck pain days over 4 weeks. Pain intensity: VAS. Disability: HIT-6 and NDI. PPT: algometry over upper trapezius trigger point. Cervical flexion–rotation test. Health-related quality of life: EQ-5D. All outcomes assessed at baseline, 4 weeks, 8 weeks and 6 months by a blinded assessor.	All three groups showed statistically significant improvements over time in CGH frequency, pain and disability, but cervical spine manipulation (cervical SMT) produced the largest and most sustained changes. At 6 months, CGH frequency decreased from 16.8 ± 1.8 to 2.9 ± 0.5 days/4 weeks in CSM, compared with 6.1 ± 0.7 in TSM and 10.8 ± 1.1 in CPT; between-group differences (CSM vs. TSM vs. CPT) were significant at 4 weeks, 8 weeks and 6 months (*p* = 0.001), with large effect sizes favoring cervical SMT. CgH pain intensity (VAS) fell to 0.8 ± 0.2 in cervical SMT vs. 1.7 ± 0.3 (TSM) and 3.7 ± 0.4 (CPT) at 6 months; HIT-6 scores decreased more in CSM (to 31.2 ± 3.8) than in TSM (39.5 ± 4.5) and CPT (48.4 ± 4.1). Neck pain frequency and intensity, CFRT right/left, NDI and EQ-5D all improved significantly in all groups, but linear mixed-model and post hoc analyses showed greater improvements and larger MCID changes in cervical SMT than TSM and CPT across all follow-ups (*p* < 0.001 for most comparisons). TSM was consistently superior to CPT but inferior to cervical SMT. No serious adverse events were reported.
Nambi et al. [38] Prospective, single-blind, parallel-group randomized controlled trial	**n = 84** Profile: Adults 18–60 years with chronic CGH >3 months, diagnosed according to ICHD-3 criteria; CGH pain intensity 3–8/10; CGH attributed to cervical spine dysfunction; reduced cervical range of motion; neck pain followed by headache; neck stiffness and movement restriction.	**SMT (n = 28)** HVLAT cervical manipulation per Peterson & Bergmann; patient supine, therapist at head, cradle hold; pre-manipulative rotation 30–45° away from pain, then HVLAT thrust toward the side of pain; sites of dysfunction identified each visit by palpation; up to two thrust attempts per session provided no new contraindications emerged. + Five min hot pack to neck/upper back before treatment; then four sessions/week for 4 weeks (total 16 sessions), delivered by experienced manual therapists; all participants also performed daily neck isometric exercises in four directions (10 s holds ×15 reps, three times/day) and were instructed to continue after 4 weeks.	**MMT (n = 28)** Patient seated; therapist standing at side, using craniocervical sustained natural apophyseal glide at C2 with ventral–cranial glide (~45°) while patient actively moves into the symptomatic direction; glide maintained for 10 s, repeated 10 times (~8 min), including end-range overpressure; Mulligan’s mobilizations applied toward side of dysfunction/pain in upper cervical segments. + Same pre-treatment hot pack and same neck isometric exercise program as other groups. **CMT (n = 28)** Fifteen min conventional soft tissue massage of cervical region using massage cream; patient supine with head supported. Circular kneading with fingertips along cervical vertebrae C7–C1 bilaterally (distal–proximal, three repetitions per level); then with head rotated, kneading along levator scapulae, sternocleidomastoid, scalenes and upper trapezius from insertion to origin on each side; finally, kneading over longissimus capitis, splenius capitis, semispinalis capitis and suboccipital muscles in centripetal direction, repeated three times. + Same pre-treatment hot pack and same neck isometric exercise program as other groups.	CGH and neck pain frequency: number of CGH and neck pain days over 4 weeks. Pain intensity: VAS. Disability: HIT-6 and NDI. PPT: algometry over upper trapezius trigger point. Cervical flexion–rotation test. Health-related quality of life: EQ-5D. All variables assessed at baseline, 4 weeks, 8 weeks and 6 months by a blinded assessor.	All groups improved over time, but Mulligan mobilization (MMT) produced the greatest and most consistent benefits, followed by SMT, with CMT showing the smallest changes. At 6 months, CGH frequency decreased from 17.2 ± 2.1 to 3.5 ± 0.6 days/4 weeks in MMT, 18.4 ± 1.9 to 5.5 ± 0.8 in SMT and 17.8 ± 1.8 to 10.8 ± 1.5 in CMT; between-group differences were significant at 4 weeks, 8 weeks and 6 months (*p* < 0.001), with the largest mean difference in favor of MMT vs. CMT (MCID reached earlier in MMT at 4 weeks). CGH pain intensity (VAS) fell to 0.7 ± 0.3 in MMT, 1.9 ± 0.4 in SMT and 3.8 ± 0.4 in CMT at 6 months; CGH disability (HIT-6), neck pain frequency and intensity, FRT right/left, NDI and EQ-5D all improved significantly in all groups, with mixed-model and post hoc analyses showing greater improvements and larger standardized mean differences for MMT vs. SMT and CMT (*p* < 0.001 for most comparisons). Neck PPTs did not differ between groups at 4 and 8 weeks but were significantly higher (better) in MMT at 6 months compared with SMT and CMT. No adverse events or serious complications were reported. Authors conclude that Mulligan’s mobilization provides superior short- and long-term outcomes for CGH compared with SMT and conventional massage, when all are combined with heat and neck isometric exercise.
McDevitt et al. [50] Randomized, repeated-measures crossover clinical trial	**n = 48** Profile: 18–65 years with CGH as primary complaint; unilateral or side-dominant headache associated with neck pain; headache aggravated by neck movement or sustained postures; joint tenderness and peripheralization into the head at ≥1 upper cervical joint (C0–C3); ≥1 headache/week over previous 2 months. Mean age 34.4 years; ~73% women; mean symptom duration >5 years.	**TSM plus thoracic mobility exercise (n = 24)** Participants attended 1–2 sessions/week for up to 4 weeks (maximum six sessions), each ~15 min. Manual therapy: prescriptive HVLAT techniques to upper, mid and lower thoracic spine and cervicothoracic junction (five standardized TSM techniques targeting T1–T12 and CT junction) performed by experienced orthopedic/manual therapists. Home program: supine thoracic mobility exercise over towel/foam roller (8–10 repetitions in up to three thoracic levels, 3–4 times/day) throughout the 4-week treatment phase and advised to continue usual activities that did not exacerbate symptoms.	**Passive control (n = 24)** During the initial 4 weeks, the Hold group received no manual therapy or exercise prescription for their headaches and continued their usual activities; this served as the control condition to isolate natural course of symptoms. At 4 weeks, groups crossed over: Hold group then received the same 4-week TSM + thoracic mobility program, while the original TSM group entered a 4-week no-treatment phase. For between-group comparisons, the key time point is 4 weeks.	Pain intensity: VAS. Disability: HDI and NDI. Perceived clinical change: GROC. Adverse events/side effects. Outcomes measured at baseline, 4, 8 and 12 weeks; 4-week point represents comparison of active TSM vs. Hold (no treatment), 8- and 12-week points reflect both groups having received TSM.	At 4 weeks (TSM vs. Hold), HDI between-group difference favored TSM but was not statistically significant (mean difference 7.39 points, 95% CI −4.39 to 19.18; *p* = 0.214). NDI showed a significant between-group difference favoring TSM (mean difference 6.90 points, 95% CI 0.05 to 13.75; *p* = 0.048), exceeding the MCID (5.5 points) for CGH. NPRS also favored TSM (mean difference 2.2 points, 95% CI 0.7 to 3.8; *p* = 0.006), exceeding MCID thresholds. Odds of achieving clinically important improvement on GRC (≥+4) were markedly higher with TSM than Hold at 4 weeks (OR ≈ 17.5, 95% CI 3.3–92.9; *p* < 0.001), with 62.5% responders in TSM vs. 8.3% in Hold. After crossover (by 8 and 12 weeks), when both groups had received TSM, between-group differences in HDI, NDI and NPRS were no longer significant, suggesting similar benefits regardless of treatment order. No serious adverse events or moderate/severe side effects were reported; TSM was well tolerated. Overall, six sessions of TSM plus thoracic mobility exercise improved neck-related disability, pain and perceived global change in chronic CGH, but did not significantly change headache-specific disability (HDI) compared with no treatment at 4 weeks.
Mousavi-Khatir et al. [43] Triple-blind randomized controlled trial with three parallel groups	**n = 69** Profile: Adults 18–60 years with CGH diagnosed by neurologist using Sjaastad & Fredriksen criteria: unilateral neck-originating pain radiating to frontotemporal region, exacerbated by neck movements, restricted CROM, and tenderness at ≥1 upper cervical joint (C1–C3). Headache at least once/week for >3 months. All had active MTrPs in ipsilateral suboccipital, upper trapezius or sternocleidomastoid muscles reproducing typical headache symptoms. Groups comparable at baseline for demographics and clinical variables.	**DN + CPT (n = 23)** Four DN sessions (second, fifth, eighth, 12th sessions) targeting active MTrPs in ipsilateral upper trapezius, suboccipital and sternocleidomastoid muscles. For upper trapezius and suboccipital muscles patients were prone; for SCM supine. Skin disinfected, 0.25 × 30 mm filiform needle inserted with guide tube into active MTrP until LTR elicited, then repeatedly moved within the MTrP to provoke multiple LTRs until they were exhausted (typically 60–90 s per point). Pragmatic approach: only MTrPs reproducing headache symptoms at each session were needled. + Fifteen CPT sessions over 5 weeks (3/week) consisting of TENS (20 min), infrared (10 min), continuous ultrasound to cervical spine (5 min, 1 MHz), plus a neck exercise program including craniocervical flexion exercises.	**Sham DN + CPT (n = 23)** Four sessions of superficial needling at points away from active MTrPs in the same muscles and sessions as DN + CPT, mimicking the DN procedure (same positioning, needle type and handling) but applied superficially without eliciting LTRs; patients were unaware whether needling was real or sham. + Same 15-session CPT protocol (TENS, infrared, ultrasound, craniocervical flexion exercises). **CPT (n = 23)** Same 15-session CPT protocol (TENS, infrared, ultrasound, craniocervical flexion exercises).	Headache intensity: NPRS. Headache frequency: number of headache days in the past week. Disability: NDI. Deep neck flexor performance: CCFT, activation score in mmHg using pressure biofeedback. Active cervical ROM: flexion, extension, rotation to affected and unaffected sides (degrees) measured with universal goniometer in standardized seated position. All variables assessed at baseline, immediately post-treatment, and at 1-, 3-, and 6-month follow-ups by a blinded assessor.	DN + PT produced significantly greater improvements than sham DN + CPT and CPT alone in most outcomes over 6 months. Repeated-measures ANOVA showed a significant Group × Time interaction for headache intensity (F = 10.89, *p* < 0.001, partial η^2^ = 0.263): DN + CPT had larger reductions at all follow-ups (e.g., NPRS from 8.1 ± 1.3 to 1.2 ± 1.2 at 6 months) than sham (7.2 ± 1.6 to 3.0 ± 1.1) or PT alone (7.6 ± 1.3 to 3.4 ± 0.7), with between-group differences vs. sham and PT ranging from −1.4 to −2.2 points (all significant) but often below the MCID of 2.5. Headache frequency showed a small but non-significant Group × Time interaction after correction (F = 2.65, *p* = 0.048, η^2^ = 0.008), although DN + CPT consistently reduced weekly headache days more than the other groups (e.g., to 0.9 ± 0.8 vs. 2.0 ± 1.0 at 6 months). For NDI, Group × Time interaction was significant and large (F = 14.41, *p* < 0.001, η^2^ = 0.321): DN + CPT showed greater disability reductions (32.0 ± 4.0 to 6.3 ± 4.8) than sham (31.4 ± 5.4 to 12.9 ± 5.2) or CPT (33.6 ± 5.2 to 13.9 ± 5.8), with between-group differences approaching the 5.5-point MCID but with wide CIs. CCFT and all CROM measures also showed significant Group × Time interactions (*p* < 0.001), with DN + CPT achieving modest but statistically greater gains in deep flexor performance and flexion, extension and rotation ROM (between-group differences ~3–13°) than both comparators. No serious adverse events were reported; post-needling soreness occurred in ~40% of DN + CPT patients, lasting 48–72 h and resolving spontaneously. Overall, adding DN to multimodal CPT yielded statistically superior, but only small-to-moderate clinically relevant improvements in pain, disability, cervical ROM and deep flexor performance compared with sham DN + CPT or CPT alone in CGH with active cervical MTrPs.
Dunning et al. [44] Randomized, single-blind, multicenter parallel-group clinical trial	**n = 142** Profile: Adults with CGH. Diagnosis according to Cervicogenic Headache International Study Group criteria: unilateral non-throbbing head pain starting in upper posterior neck/occipital region and spreading to oculofrontotemporal area; pain provoked by neck movement/sustained positions; restricted cervical ROM (≤32° rotation on flexion–rotation test); pain on palpation of ≥1 upper cervical joint (C0–C3). Mean age ≈ 40 years; symptom duration ≈ 4.5 years; moderate baseline pain and disability.	**Perineural electrical DN + SMT (n = 74)** Up to eight sessions over 4 weeks (1–2/week). Manual therapy: HVLAT directed primarily to upper cervical (C1–C2) and upper thoracic (T1–T2) segments using standardized techniques. + Semistandardized protocol of 8–12 needles including 6–8 occipito-cervical points (suboccipital muscles, periosteal/perineural regions around greater/lesser occipital and third occipital nerves), one distal point in ipsilateral hand, and up to five oculofrontotemporal points (supraorbital/ophthalmic branch) ± up to four upper thoracic paraspinal points. Sterile single-use needles (0.18 × 15, 0.25 × 30, 0.30 × 40 mm) inserted intramuscularly/periosteally/perineurally; after eliciting typical needling sensations (aching, tingling, heaviness), needles retained 20 min with low-frequency (2 Hz) biphasic continuous electrostimulation to up to eight needles at a “mild–moderate” intensity.	**Cervical and upper thoracic non-thrust mobilization + exercise (n = 68)** Same 4-week schedule (up to eight sessions). Manual therapy: non-thrust, slow oscillatory mobilization techniques directed to upper cervical (C1–C2) and upper thoracic (T1–T2) joints using Maitland-style graded mobilizations. + Supervised craniocervical flexion exercises (deep neck flexor training with pressure biofeedback) and progressive periscapular resistance exercises; dosage and progression individualized based on symptom response and tolerance (progression only if symptoms decreased and soreness did not last > a few hours). No DN or spinal thrust manipulation was applied.	Headache pain intensity: NPRS. Headache frequency: number of headache days in past week. Headache duration: total headache hours in past week categorized 0–5, 6–10, 11–15, 16–20, 21–25, ≥26 h. Disability: NDI. Perceived clinical change: GROC. Outcomes measured at baseline, 1 week, 4 weeks and 3 months after first session by blinded assessors.	Group × Time interaction was significant for headache intensity (NPRS: F = 23.464, *p* < 0.001), headache frequency (F = 13.407, *p* < 0.001) and disability (NDI: F = 10.702, *p* < 0.001), all favoring SMT + eDN at 3 months. At 3 months, between-group mean difference in NPRS change was −2.9 points (95% CI −3.5 to −2.3; *p* < 0.001), exceeding the MCID; effect size large (SMD ≈ 1.25). NDI between-group difference in change was −8.8 points (95% CI −11.4 to −6.0; *p* < 0.001), also exceeding the MCID and yielding a large effect (SMD ≈ 0.94). Headache frequency decreased more in SMT + eDN (mean change −3.4 vs. −1.5 days/week; between-group difference −1.8, 95% CI −2.5 to −1.4; *p* < 0.001; large SMD ≈ 0.97), and headache duration categories shifted to shorter time ranges compared with comparator group (*p* < 0.001). At 3 months, 66% of SMT + eDN patients vs. 21% of comparator group had completely stopped headache medication (χ^2^ = 29.889, *p* < 0.001). Based on GROC ≥+5, 77% in SMT + eDN vs. 15% in comparator group achieved a successful outcome (χ^2^ = 54.840, *p* < 0.001; NNT ≈ 1.6). No serious adverse events were reported; in the SMT + eDN group ~61% had transient post-needling soreness and ~24% mild bruising, resolving spontaneously within days. Authors conclude that upper cervical/thoracic thrust manipulation plus electrical DN is superior to non-thrust mobilization and exercise for short- to mid-term improvement in pain, disability, headache burden and medication use in CH.
Rist et al. [49] Pilot randomized controlled trial, two parallel groups	**n = 61** Profile: Adult women aged 20–55 years with diagnosis of episodic migraine (with/without aura) according to ICHD-3, migraine history ≥1 year, and 4–13 migraine days during a 4-week run-in (confirmed by daily migraine logs). Mean age 36.4 years; mean migraine frequency during run-in 7.6 ± 2.2 days/month; majority with typical migraine features (unilateral, pulsating pain, photophobia/phonophobia, nausea, activity avoidance).	**MCC+ EUC (n = 29)** Up to 10 sessions of individualized multimodal chiropractic care over 14 weeks at an integrative medicine clinic. First visit: detailed physical examination; patients classified into diagnostic categories with pre-specified but flexible treatment algorithms. Care was tailored to patient findings and preferences and could include: SM and joint mobilization (cervical/thoracic/temporomandibular joint as indicated), soft tissue relaxation/release techniques, posture correction and spinal stabilization/strengthening exercises, stretching, relaxation techniques, ergonomic advice and education. Participants could opt out of any component (e.g., manipulation). + Usual medical care as prescribed by their physicians plus standardized migraine education materials (American Headache Society information on migraine pathophysiology, triggers, treatments and comorbidities).	**EUC (n = 32)** Same usual medical care as described.	Feasibility of participant recruitment. Retention and adherence to protocol. Evaluation of adverse effects. Number, intensity, and duration of migraine days. Amount of medication used. Migraine Disability Assessment: MIDAS. Disability: HIT-6. Migraine-Specific Quality of Life: MSQL. Outcomes calculated for run-in (baseline), weeks 11–14 (“initial follow-up”), and the 4-week post-intervention phase (“final follow-up”).	Feasibility: recruitment was slower than planned (61 randomized over 20 months) and only 74% completed all assessments, so prespecified feasibility criteria were not fully met, but retention at main follow-up points was good (≈84–97%) and adherence to chiropractic sessions was high (264/290 visits attended; 83% attended ≥75% of sessions). Safety: Ninety-eight non-serious adverse events (AEs) were reported (39 in EUC, 59 in MCC + EUC); no serious AEs occurred. In MCC + EUC, 15 events in four participants were “possibly related” and five in 5 participants “related” to treatment (mostly transient musculoskeletal stiffness or migraine); no related AEs in EUC alone. Clinical effects: compared with EUC, MCC + EUC produced greater reductions in migraine days from run-in to weeks 11–14 (mean change −2.90 vs. −0.98 days; between-group difference −1.92; 95% CI −3.46 to −0.37), an effect that persisted at final follow-up. MCC + EUC also showed larger improvements in migraine severity, medication use and duration (between-group differences in changes generally favored MCC + EUC but some CIs included 0). Disability and impact: greater decreases in MIDAS (between-group difference in change −5.58; 95% CI −10.44 to −0.72) and HIT-6 (−3.62; 95% CI −6.52 to −0.73) at week 14 in MCC + EUC; MSQ role-restriction and emotional function domains improved slightly more with MCC + EUC, though effect sizes were small. Responder rates (≥50% reduction in migraine days) were higher with MCC + EUC (10 vs. seven responders at initial follow-up; OR ≈ 2.17, 95% CI 0.68–6.95; similar pattern at final follow-up), but CIs were wide.
Corum et al. [45] Prospective randomized controlled trial, three parallel groups	**n = 39** Profile: Adults 19–48 years with TTH (episodic or chronic) and neck pain, diagnosed according to ICHD-3 beta criteria (bilateral, pressing/tightening, mild–moderate intensity, no aggravation with physical activity, no nausea/vomiting/photophobia/phonophobia). Symptoms >3 months and average pain ≥3/10 on VAS in previous week; at least one upper cervical segmental dysfunction (functional and pain-provocation tests).	**SMT+ exercise (n = 12)** Eight sessions over 4 weeks (two/week) of segment-specific HVLAT manipulation to dysfunctional upper cervical segments. Patient seated; physician’s hypothenar on mastoid, middle finger on upper articular pillar; cervical spine placed in slight flexion and 15–20° lateral flexion without rotation; diagnostic mobilization to take up slack and exclude contraindications, then dorsal-to-ventral HVLAT thrust. + Combined with home exercise program ≥3 days/week: 20–30-min sessions including cervical ROM warm-up/cool-down, stretching of cervical/upper thoracic muscles (trapezius, levator scapulae, sternocleidomastoid), and strengthening (cervical isometrics and concentric deep cervical flexor exercises), three sets of 5–10 reps, with 30–60-s rests; adherence monitored via exercise diary and post-session checks.	**Myofascial release + exercise (n = 15)** Eight sessions over 4 weeks of suboccipital inhibition. Patient supine; therapist seated at head, fingers in suboccipital region applying deep, progressive inhibitory pressure perpendicular to the insertions of neck extensor muscles in the occiput while thumbs stabilize the head; pressure maintained ~10 min until reduction in muscle tone and tissue “release”. + Same exercise program. **Exercise (n = 12)** Same education and home exercise program as intervention groups (cervical ROM, stretching of cervical/upper thoracic muscles, deep cervical flexor and isometric strengthening; 20–30 min, ≥3 days/week, with diary monitoring). No manual therapy (no manipulation or suboccipital inhibition).	Headache frequency: number of days with headache in previous 2 weeks (days/2 weeks), recorded in a headache diary. Headache and neck pain intensity: VAS Disability: HIT-6 and NDI. PPT: temporalis anterior muscle, measured with mechanical algometer. All outcomes measured at baseline, post-treatment (≥72 h after last session) and 3-month follow-up by an assessor blinded to group allocation.	Manipulation + exercise produced significant improvements in all outcomes: headache frequency, VAS headache, VAS neck pain, HIT-6, NDI and PPT increased significantly post-treatment and at 3-month follow-up (e.g., headache frequency −3.3 ± 1.2 and −3.0 ± 2.1 days/2 weeks; VAS headache −3.8 ± 1.5 and −3.5 ± 2.1; HIT-6 −10.1 ± 6.1 and −7.4 ± 9.1; NDI −6.5 ± 4.5 and −8.3 ± 6.1; PPT +0.7 ± 0.8 and +1.0 ± 0.7; all *p* ≤ 0.041). Myofascial release + exercise significantly reduced headache frequency, headache and neck pain intensity, HIT-6 and NDI only immediately post-treatment, with smaller changes and partial loss of effect at 3 months; PPT did not change significantly. Control (exercise only) showed no significant changes in any outcome. Manipulation was consistently superior to control, and often to myofascial release. Compared with control, manipulation achieved greater reductions in headache frequency post-treatment and at 3 months (*p* < 0.001 and *p* = 0.001), VAS headache (*p* < 0.001 and *p* = 0.002), VAS neck pain (*p* = 0.001 and *p* = 0.014), and NDI (*p* < 0.001 and *p* = 0.007). HIT-6 improvement was significantly greater in manipulation vs. control post-treatment (*p* < 0.001). Compared with myofascial release, manipulation showed larger improvements in headache frequency at 3 months (*p* = 0.001), VAS headache at 3 months (*p* = 0.014), NDI post-treatment and at 3 months (*p* = 0.021 and *p* = 0.028), and PPT post-treatment and at 3 months (*p* = 0.003 and *p* = 0.002). Clinically, only the manipulation group exceeded the MCID thresholds for HIT-6 (−10.1 points post-treatment), NDI (−8.3 points at 3 months), and VAS neck pain (−2.8 to −3.0 vs. MCID 1.3). Authors conclude that upper cervical HVLA manipulation combined with exercise is more effective than suboccipital inhibition plus exercise and exercise alone in reducing headache frequency and intensity, neck pain and disability, and in increasing temporalis PPT in TTH patients with neck pain.
Lerner-Lentz et al. [46] Pragmatic randomized clinical trial, two parallel groups	**n = 45** Profile: Adults 18–65 years with CGH (unilateral headache associated with neck pain, aggravated by neck postures/movement, and tenderness in at least one upper cervical joint C0–C3). Required ≥2 headaches in the last month, NDI ≥20% (≥10/50) and pain intensity ≥2/10 on NPRS.	**SMT (n = 21)** Patient prone; therapist assessed C1–C3 using central posterior–anterior (CPA) force over spinous process of C2–C3 and unilateral posterior–anterior forces over articular pillar/lamina of C2–C3 and lateral mass of C1 to identify the level reproducing the patient’s comparable sign. At the identified level, therapists applied a single high-velocity low-amplitude manipulation at end range, chosen pragmatically as either: (1) localized cervical rotation manipulation (supine, chin–jaw hold, rotation and side-bending to lock all three planes followed by HVLAT toward the symptomatic side); or (2) longitudinal cephalad C1–C2 traction manipulation (supine, mastoid hook with thrusting knuckle, cranially directed HVLAT traction). Only one manipulation was performed at the target segment per treatment session. + A standardized home exercise program (chin tucks, supine chin nods, scapular depression and retraction exercises with progressive resistance), with prescribed sets/reps several times per day and adherence logged.	**Mobilization (n = 24)** Same examination and identification of the most symptomatic upper cervical segment. Once the symptomatic level was identified, mobilization was applied at that segment for 30 s using smooth, rhythmical oscillations, then repeated twice more (3 × 30 s total). Mobilization directed pragmatically based on clinical findings. + Same home exercise program.	Disability: NDI and HIT-6. Pain intensity: NPRS. Active cervical range of motion (ACROM: flexion, extension, right/left side-bending, right/left rotation) measured with inclinometer and cervical ROM device. Perceived clinical change: GROC. PASS: yes/no “current state satisfactory?”. Side effects: type and duration. NDI, NPRS, HIT-6, GRC and PASS at baseline, 48 h, discharge and 1-month follow-up.	There were no significant between-group differences for the primary or secondary outcomes. Mixed-model ANOVA showed a non-significant group × time interaction for NDI (*p* = 0.91, partial η^2^ = 0.013), NPRS (*p* = 0.81) and HIT-6 (*p* = 0.89), as well as for all ACROM measures (flexion, extension, side-bending, rotation; all *p* > 0.65). Both groups demonstrated statistically significant within-group improvements over time in disability, pain, headache impact and ACROM (*p* < 0.05), with similar magnitudes of change. At 1-month follow-up, mean NDI change from baseline was −12.8 (SD 9.2) in the mobilization group and −13.9 (SD 8.0) in the manipulation group (between-group difference −1.1; 95% CI −5.6 to 3.4; *p* = 0.68). NPRS change was −3.4 (SD 2.6) vs. −4.5 (SD 2.2) (difference −0.98; 95% CI −2.4 to 0.45; *p* = 0.18). HIT-6 change was −11.9 (SD 7.9) vs. −12.6 (SD 7.3) (difference −0.67; 95% CI −5.3 to 3.9; *p* = 0.77). GRC scores were slightly higher in the manipulation group at 48 h, discharge and 1 month, but between-group differences were not significant after correction for multiple testing (e.g., 1-month GRC 4.9 vs. 5.8; *p* = 0.07). PASS success rates were high and similar between groups (discharge: 92% vs. 95%; 1 month: 87.5% vs. 100%; both *p* > 0.20). No adverse side effects were reported in either group. Authors conclude that, when applied pragmatically and combined with a standardized home exercise program, upper cervical manipulation and mobilization yield comparable improvements in pain, disability and headache impact in patients with CGH.
Rezaeian et al. [47] Randomized, placebo-controlled trial	**n = 40** Profile: Adults 25–55 years with migraine diagnosed according to International Headache Society criteria, screened at a neurology clinic. All had active MTrPs in the SCM muscle reproducing their typical migraine pain (tender spot with referred pain and jump sign). Groups were comparable at baseline in age, sex, BMI and headache characteristics.	**DN (n = 20)** Three treatment sessions over 1 week at 48 h intervals. Patient supine with neck slightly laterally flexed toward the symptomatic side; active SCM MTrPs palpated by pincer grip after locating the carotid artery. Standard sterile acupuncture needles (0.25 × 25 mm) inserted with guide tube into the muscle belly of both sternal and clavicular heads, directed antero-posteriorly to avoid neurovascular structures. Between eight and 10 fast, deep insertions were performed into each MTrP (Hong technique) to elicit local twitch responses; after needle removal, firm compression was applied for 90 s to reduce post-needling soreness.	**Sham DN (n = 20)** Same patient positioning, skin preparation and MTrP identification as DN group. A blunted needle within a guide tube was pressed against the skin over the SCM MTrP to create a pricking sensation without skin penetration. The same pressure, contact time and number of “applications” (three sessions over 1 week, 48 h intervals) were used, but no actual needling of the muscle occurred.	Headache frequency: number of days with headache over the previous 2 weeks, recorded in a daily headache diary. Headache intensity: 0–5 ordinal scale (0 = no pain, 5 = maximum pain, unable to do anything). Headache duration: hours of headache per day. Drug consumption: number of pain-relieving tablets used on headache days. PPT over SCM MTrP: measured with an electronic algometer (three trials, mean value). SCM muscle thickness: measured with B-mode ultrasound at the mid-belly trigger point. Active cervical ROM: flexion, extension, right/left rotation, right/left lateral flexion, measured with a goniometer. All variables assessed at baseline (2-week run-in), immediately after the 1-week intervention, and at 1-month follow-up.	Compared with placebo, DN produced significantly greater improvements in all headache-related outcomes. In the DN group, headache frequency, intensity, duration, and drug consumption all decreased significantly immediately post-treatment and at 1-month follow-up (all *p* < 0.001), whereas no meaningful changes occurred in the placebo group. Between-group differences at 1 month favored DN for headache frequency (mean difference −2.05 days), intensity (−1.35 points), duration (−19.35 h), and drug consumption (−4.25 tablets) (all, *p* < 0.001), with large effect sizes (Cohen’s d = −2.48, −2.09, −2.25, and −4.02, respectively). PPT over the SCM MTrP also favored DN after treatment (mean difference 0.96 kg/cm^2^; *p* = 0.006) and at 1 month (2.29 kg/cm^2^; *p* < 0.001; d = 2.19), while SCM muscle thickness showed significant between-group differences favoring DN both post-treatment and at follow-up (1-month mean difference 1.77 mm; *p* < 0.001; d = 2.75). Active cervical ROM in all directions increased in the DN group and decreased in controls, with all between-group comparisons significant at follow-up (all *p* < 0.001) and very large effect sizes (d = 3.42–5.46). Overall, three sessions of DN into SCM MTrPs led to clinically and statistically significant reductions in migraine headache burden and medication use, along with increased PPT, muscle thickness, and cervical mobility, compared with placebo needling.
Togha et al. [48] Randomized controlled trial, three parallel groups	**n = 29** Profile: Adult women with CGH originating from an active MTrP in the SCM muscle; diagnosis made by an expert neurologist according to ICHD-3 criteria. Required a single active SCM MTrP reproducing the usual headache pattern (taut band, hypersensitive spot, local twitch response, and typical referred pain). Mean age 35.3 ± 12.2 years.	**DN (n = 10)** Four treatment sessions within 8 days (one-day intervals). Subjects supine with neutral head; SCM taut band grasped between thumb, index and middle fingers; a 0.25 × 40 mm filiform needle repeatedly inserted in an antero–posterior direction into the active MTrP until local twitch responses were exhausted.	**IC (n = 9)** Four sessions within 8 days. Subjects supine with head rotated contralaterally; SCM taut band grasped between thumb and index finger; maximal tolerable digital pressure applied directly over the MTrP for 30–60 s, repeated three times with 30 s intervals; treatment stopped if headache pattern was reproduced or pain resolved. **Passive control (n = 9)** No therapeutic intervention during the study period.	Headache intensity: VAS. Headache frequency: number of headache days per 2-week period. Headache duration: hours of headache per day. PPT: SCM MTrP using digital algometer. MTrP elastic modulus. MTrP area (mm^2^) using B-mode ultrasound with stress–strain analysis (ImageJ). All variables recorded over a 2-week run-in period (baseline) and again for the 2 weeks following the final treatment session (post-treatment).	Both DN and IC groups showed significant improvements versus control in all clinical headache outcomes and PPT. Compared with baseline, DN produced significant reductions in headache intensity (−3.01; 95% CI −3.79 to −2.22), frequency (−3.00 days/2 weeks; 95% CI −3.95 to −2.04), and duration (−6.15 h/day; 95% CI −7.96 to −4.34), while IC also reduced headache intensity (−2.38; 95% CI −3.92 to −0.94), frequency (−2.22 days/2 weeks; 95% CI −3.22 to −1.22), and duration (−5.04 h/day; 95% CI −9.19 to −0.88). In contrast, the control group showed no meaningful changes. Inter-group comparisons confirmed significant differences versus control for both DN and IC, with effect direction favoring both active interventions over no treatment: for DN versus control, the between-group differences were 3.14 points for headache intensity (95% CI 2.30 to 3.96; *p* < 0.001), 2.90 days/2 weeks for headache frequency (95% CI 1.26 to 4.53; *p* < 0.001), and 6.46 h/day for headache duration (95% CI 4.52 to 8.41; *p* = 0.002); for IC versus control, the corresponding differences were 2.55 points (95% CI 0.80 to 4.30; *p* = 0.001), 2.11 days/2 weeks (95% CI 0.46 to 3.76; *p* = 0.007), and 5.36 h/day (95% CI 0.75 to 9.69; *p* = 0.015), respectively. However, no significant differences were found between DN and IC for headache intensity, frequency, or duration (all *p* = 1.000 or *p* = 0.800). PPT also improved in both intervention groups, with within-group changes of 1.74 kg/cm^2^ (95% CI 1.00 to 2.49) for DN and 2.32 kg/cm^2^ (95% CI 1.13 to 3.50) for IC; compared with control, the between-group differences were 1.05 kg/cm^2^ for DN (95% CI −0.27 to 2.38; *p* = 0.032) and 1.63 kg/cm^2^ for IC (95% CI 0.06 to 2.95; *p* = 0.039), with no significant difference between DN and IC (*p* = 0.661). For biomechanical ultrasound measures, MTrP elastic modulus and RIE decreased in both DN and IC, but without significant between-group differences versus control. By contrast, MTrP area decreased significantly in both intervention groups compared with control, with between-group differences of 2.67 mm^2^ for DN (95% CI 1.48 to 3.86; *p* = 0.002) and 1.87 mm^2^ for IC (95% CI 0.06 to 3.67; *p* = 0.042), again with no significant difference between DN and IC (*p* = 0.815). A significant positive correlation was found between headache intensity and MTrP elastic modulus (r = 0.490; *p* = 0.007), suggesting that stiffer MTrPs are associated with more intense headache. Overall, both DN and IC showed short-term clinically relevant improvements versus control, but neither technique was clearly superior to the other.

AROM: active range of movement; CFRT: cervical flexion–rotation test; CGH: cervicogenic headache; CI: confidence interval; CMT: conventional massage therapy; CPT: conventional physiotherapy; CROM: cervical range of movement; DN: dry needling; EUC: enhanced usual care; FRT: flexion–rotation test; GROC: global rating of change; HVLAT: high-velocity low-amplitude thrust; IC: ischemic compression; ISM: instrument-assisted soft tissue mobilization; MCC: multimodal chiropractic care; MDC: minimal detectable change; MCID: minimal clinically important difference; MMT: Mulligan mobilization therapy; MTrP: myofascial trigger point; NPRS: numeric pain rating scale; PASS: patient acceptable symptom state; SCM: sternocleidomastoid; PPT: pressure pain threshold; RIE: relative index of elasticity; SMT: spinal manipulative therapy; TENS: transcutaneous electrical nerve stimulation; TTH: tension-type headache; VAS: visual analogue scale.

## Data Availability

The original data presented in the study are openly available in the Open Science Framework platform (https://doi.org/10.17605/OSF.IO/2JXZN, updated 30 October 2025).

## Supplementary Materials

The following supporting information can be downloaded at: https://www.mdpi.com/article/10.3390/jcm15052084/s1, Table S1: PRISMA checklist.
